# A dataset on affiliation of venture capitalists in China between 2000 and 2016

**DOI:** 10.1038/s41597-021-00993-w

**Published:** 2021-08-04

**Authors:** Jin Chen, Tianyuan Chen, Yifei Song, Bin Hao, Ling Ma

**Affiliations:** 1 Nottingham University Business School China, University of Nottingham Ningbo China, Ningbo, China; 2 UNNC-NFTZ Blockchain Laboratory, Ningbo, China; 3 School of Business, East China University of Science and Technology, Shanghai, China

**Keywords:** Business, Economics, Government

## Abstract

Prior literature emphasizes the distinct roles of differently affiliated venture capitalists (VCs) in nurturing innovation and entrepreneurship. Although China has become the second largest VC market in the world, the unavailability of high-quality datasets on VC affiliation in China’s market hinders such research efforts. To fill up this important gap, we compiled a new panel dataset of VC affiliation in China’s market from multiple data sources. Specifically, we drew on a list of 6,553 VCs that have invested in China between 2000 and 2016 from CVSource database, collected VC’s shareholder information from public sources, and developed a multi-stage procedure to label each VC as the following types: GVC (public agency-affiliated, state-owned enterprise-affiliated), CVC (corporate VC), IVC (independent VC), BVC (bank-affiliated VC), FVC (financial/non-bank-affiliated VC), UVC (university endowment/spin-out unit), and PenVC (pension-affiliated VC). We also denoted whether a VC has foreign background. This dataset helps researchers conduct more nuanced investigations into the investment behaviors of different VCs and their distinct impacts on innovation and entrepreneurship in China’s context.

## Abstract (summary)

**Table:** 

Measurement(s)	venture capitalist • Affiliation
Technology Type(s)	digital curation
Factor Type(s)	year
Sample Characteristic - Location	China

Machine-accessible metadata file describing the reported data: 10.6084/m9.figshare.14811009

## Background & Summary

Prior literature suggests distinct roles of differently affiliated venture capitalists (VCs) (e.g., independent, corporate, and governmental VCs) in nurturing innovation and entrepreneurship. A VC’s affiliation determines the foci of its attention and investment logic, both of which shape its strategic choices in terms of industry, syndication partners, and portfolio ventures^1^. Such selection effects caused by VC affiliation, together with unique resources endowed by each type of affiliation, can be further carried over to portfolio ventures and affect those ventures’ innovation, growth, and financial performance^2,3^. While scholars in the U.S. or European contexts have increasingly realized the need to differentiate VCs by affiliation, research on the second-largest VC market – China’s VC market^4^ – has paid relatively less attention to this either in theoretical development or in empirical investigation, due to the lack of information about VC affiliation in this setting.

Supplying the data of VC affiliation in China’s market is of great importance for two reasons. First, recent research on innovation and entrepreneurship has recognized the importance of institutional variation to VC investment strategies and venture performance^1,5^. Including China’s market into this stream of research would enlarge institutional variation and enhance our understanding of how varied institutions affect different VCs’ investment strategies and behaviors and how they influence portfolio ventures.

Second, research is beginning to show strong interests in new theoretical lenses contextualized to emerging economies and associated policy implications. A hallmark of emerging economies is the strong contrast between their institutional voids and the high-velocity market and technological development. Among the emerging economies, China is the largest one and is unique due to its great power of government in intervening market development^6^. This visible hand has penetrated the VC market when China started to shift the emphasis of its innovation policies from government subsidy to governmental VCs (GVCs) in 2010s. Scholars are turning increasingly to the questions of how government interventions (e.g., in the form of GVCs) affect other types of VCs as well as entrepreneurial financing in emerging economies like China, what the outcomes are, and why.

Despite the importance to examine VC affiliation in China, prior research that focused on this context is very limited, as observed by Huang and Tian^4^ and Bruton and Ahlstrom^6^. Specifically, recent studies in China’s context have come to realize that the classification of VCs in extant databases is lacking (i.e., no information on whether a VC is independent VC, GVC, corporate VC or others). Some databases (e.g., PEdata and CVSource which are two largest databases about VC investments in China), although providing some information of VC’s state background, do not have information about VC affiliation. In addition, based on the PEdata’s sample between 1995 and 2011, Zhang^7^ found that “PEdata has a lot of missing data in recognizing whether a VC firm has a state background” and that “[among] 474 VC firms categorized by PEdata as ‘with state background’, 254 are identified as GVCs after crosschecking” against the official websites of VC firms and government agencies. The research on VC affiliation in China’s context has therefore largely been limited due to the unavailability of high-quality datasets.

In this study, we compiled a new panel dataset of VC Affiliation in China’s market (hereafter VCAC dataset) based on information from multiple sources. In particular, we gathered a list of VCs that have invested in China between 2000 and 2016 from CVSource, collected the annual information of their shareholders from public sources (e.g., Tianyancha, Qichacha, Bloomberg, and Crunchbase), and developed a multi-stage procedure to label each VC in a year as the following types: GVC (including public agency-affiliated VC and state-owned enterprise-affiliated VC), CVC (corporate VC), IVC (independent VC), BVC (bank-affiliated VC), FVC (financial/non-bank-affiliated VC), UVC (university endowment/spin-out unit), and PenVC (pension-affiliated VC). In addition, we denoted whether a VC has foreign background. This VCAC dataset can provide a fertile ground for developing contextualized theories in emerging economies that explain interactions among government, investors, and young firms^8^. The adoption of this dataset will help future studies reduce repetitive efforts in data collection and facilitate their investigation into the impact of VC affiliation on innovation and entrepreneurship in China’s emerging market. In addition, scholars in other emerging markets can apply the methodology developed in this study to their countries to classify their VC affiliations, which helps develop contextualized theories in the field of innovation and entrepreneurship.

## Methods

Our methodology to develop the VCAC dataset is grounded in existing practices and composed of two parts: typology of VC affiliation, and classification process. Specifically, we started with Chahine *et al*.’s^1^ typology of VC affiliation: *GVC (public agency-affiliated VC), CVC, IVC, BVC, FVC, UVC*, and *PenVC*. Furthermore, given the salient government intervention in China’s economy via state-owned enterprises (SOEs), we created a new type, i.e., *GVC (SOE-affiliated VC)* to label such affiliations. In addition, we added one more label termed *foreign VC* to denote whether a VC is foreign, considering potential research interests on cross-border VC investment. Noteworthy, the above types of VC affiliation may not be mutually exclusive; in practice, a VC could be labelled by more than one type. For example, SOE-affiliated VC could also be categorized as CVC if satisfying the CVC criteria in the literature. And all PenVCs belong to foreign VCs, because pension in China is centrally managed by public authorities instead of private firms.

As regards classification process, we followed prior research whenever possible and made adaptation when necessary. For example, scholars often start with a list of VCs from existing databases (e.g., VentureXpert) and manually collect information of VC affiliation from public sources (e.g., Factiva, Google, Lexus/Nexus) to see if a VC can be classified as a particular type^2,9^. Drawing on their procedure but adapting it to the context of China, we developed a multiple-stage procedure, as described in Section “Data coding scheme”.

### Data collection

The VCAC dataset compiled in this project is based on the population of investment deals in China between 2000 and 2016 that were recorded in CVSource database, which is one of the largest and most reliable databases of China’s investment deals and has been popular among previous studies^10,11^. According to CVSource, there were 6,553 VCs that have invested in China between 2000 and 2016, and according to our manual check, 6,379 unique VCs were identified. Information of VCs that was used in data processing (e.g., VC name, age, location, records of investments and portfolio ventures, and syndication partners) was collected from CVSource.

Information of VC affiliation (e.g., shareholder name, equity ratio, year) was gathered from public sources such as Tianyancha (Tianyancha.com), Qichacha (Qichacha.com), Bloomberg, and Crunchbase. Specifically, we searched information for domestic VCs mainly from Tianyancha and Qichacha (the two largest databases on Chinese business registration information), and gathered information for foreign VCs mainly from their official websites, Bloomberg, and Crunchbase. For those VCs that were not captured by the above sources, we checked media reports to gather information. Moreover, if information was inconsistent across sources, we followed Bertoni and Martí^12^ and triangulated the data with additional information available from all potential public sources (e.g., VC websites, press releases, media reports, and IPO prospectuses).

### Data coding scheme

In this section, we describe the scheme of data coding, i.e., how we developed a multiple-step procedure to identify each VC’s affiliation.

#### Step 1. Coding on shareholder type

Based on the sampling frame of VC list from CVSource, we gathered each VC’s shareholder information (shareholder name and equity) from above mentioned public sources. We call those shareholders “1^st^-level shareholders”. In total, for the 6,379 VCs, we gathered information of 22,757 1^st^-level unique shareholder-year records, and developed a three-layered coding scheme for shareholders.

#### Layer-I coding on shareholder business scope

For each 1^st^-level shareholder in a given year, based on its main business, we developed a coding scheme to classify it to one of the following mutually-exclusive types: public agency, professional, corporate, bank, financial, university, person, and pension. If information of main business is unavailable, we used a catch-all rule and classified the 1^st^-level shareholder to a ninth type as unknown.

##### Type 1. 1^st^-level shareholder as public agency type

A 1^st^-level shareholder of a VC is labelled as such when the shareholder per se is a public authority or a not-for-profit organization whose executives are designated by government. Here, the scope of public authorities ranges from central government to town government, including street offices. Village committees are excluded from public authorities because villages belong to self-governance organizations in China.

##### Type 2. 1^st^-level shareholder as professional type

A 1^st^-level shareholder of a VC is labelled as such when the main business of the shareholder is VC investment and/or VC-related services, including, but not limited to, VC investment consulting.

##### Type 3. 1^st^-level shareholder as corporate type

A 1^st^-level shareholder of a VC is labelled as such when the main business of the shareholder is providing products and services other than investment, financial or bank business. Examples of a corporate’s main business include, but not limited to, manufacturing, retailing, trading, importing, exporting, IT service, and business consulting.

##### Type 4. 1^st^-level shareholder as bank type

A 1^st^-level shareholder of a VC is labelled as such when the shareholder per se is a commercial bank (including branches).

##### Type 5. 1^st^-level shareholder as financial type

A 1^st^-level shareholder of a VC is labelled as such when the shareholder per se is a financial institute (including branches). Here, financial institutes refer to institutes that operate financial activities, including, but not limited to, securities, insurance, trust, hedge, non-performing asset management, and mortgage.

##### Type 6. 1^st^-level shareholder as university type

A 1^st^-level shareholder of a VC is labelled as such when the shareholder per se is a university or college that provides higher education.

##### Type 7. 1^st^-level shareholder as person type

A 1^st^-level shareholder of a VC is labelled as such when the shareholder per se is a natural person.

##### Type 8. 1^st^-level shareholder as pension type

A 1^st^-level shareholder of a VC is labelled as such when the shareholder per se is a pension fund.

##### Type 9. 1^st^-level shareholder as unknown type

A 1^st^-level shareholder of a VC is labelled as such when no information can be found to confirm its type as above.

#### Layer-II coding on shareholder’s SOE nature

Second, to account for the complex situation in China where government uses equity control of enterprises to intervene in the economy, we further added a second layer coding to classify whether a 1^st^-level shareholder is SOE. Noteworthy, this second layer coding of SOE is not mutually exclusive with the Layer-I coding. Specifically, a professional, corporate, bank, or financial 1^st^-level shareholder can also be labelled as SOE if satisfying the following criteria. In contrast, public agency, university, person, pension, and unknown types do not need such a second layer coding.

##### 1^st^-level shareholder as SOE type

A 1^st^-level shareholder of a VC, if it is registered in China, is labelled as SOE according to the “Measures for the Supervision and Administration of the Transactions of State-owned Assets of Enterprises (Order No. 32)” and “Measures for the Supervision and Administration of State-owned Equity of Listed Companies (Order No. 36)”. Specifically, combining No. 32 and No. 36, we define an SOE if the enterprise or unit meets one of the following circumstances: (1) solely or wholly state-owned enterprises or units; (2) an enterprise or unit whose equity ratio by solely or wholly SOEs or public authorities exceeds 50%, and one of the above solely or wholly SOEs or public authorities is the largest shareholder in the enterprise; and (3) all subsidiary enterprises or units that are above 50% owned by any enterprise or unit mentioned in the first and second circumstances. Considering the practice in VC industries, we relaxed criterion (2) to categorize SOE. According to the “Notice of the State Council on Issuing the Implementation Plan for Transferring Part of State-owned Capital to Fortify Social Security Funds”, if the state-owned equity in an enterprise exceeds 50%, 10% of its state-owned equity and associated dividends will be transferred to the National Council for Social Security Fund. Therefore, we revised criterion (2) to be: if an enterprise or unit whose equity ratio by solely or wholly SOEs or public authorities is close to 50%, and one of the above solely or wholly SOEs or public authorities is the largest shareholder in the enterprise. Instead of pre-setting the range, we manually checked those cases close to 50% and confirmed some as SOEs (ranging from 43% to 49%, and case by case judgement).

Apart from Chinese organizations, we coded a foreign 1^st^-level shareholder as SOE when it is likewise affiliated with public agencies or state-controlled enterprises in other countries.

#### Layer-III coding on shareholder foreign background

Third, given cross-border VC investment to China’s market, we added a third layer coding to classify whether a 1^st^-level shareholder is foreign, using the following rule. Noteworthy, this third layer coding of foreign is not mutually exclusive with either Layer-I or Layer-II coding. For example, a foreign 1^st^-level shareholder can also be labelled as one particular type under Layer-I coding, or as SOE under Layer-II coding. And all pension-typed 1^st^-level shareholder appeared to be foreign because China does not designate private organizations to manage pension funds.

##### 1st-level shareholder as foreign type

A 1^st^-level shareholder of a VC is labelled as foreign when (1) the shareholder has not registered in China or not originated in China, (2) the shareholder has registered in China but it is solely or wholly owned by foreign entities, or (3) the shareholder is a natural person whose name is not Chinese.

#### Step 2. Coding on VC type

Following prior studies^1^, if a VC has information on its 1^st^-level shareholders, we used the type of 1^st^-level shareholders as VC’s direct affiliation. If the information of its 1^st^-level shareholders is unavailable, we collected information of the VC’s background information and business scope and directly judged its affiliation using similar criteria that applied to 1^st^-level shareholders. Specifically, we created two modes of coding to cater different needs of scholars.

#### Non-exclusive VC type coding

According to resource-based view, a VC can have access to multiple types of resources if it has multiple parents (e.g., a parent being public agency, SOE, or corporate). In this regard, a VC can be identified as multiple affiliations. To achieve this, we developed a non-exclusive coding scheme of VC type, using the following procedures.

GVC: Based on prior literature^13^ and our interviews with industry experts, GVCs in China include two sub-types (i.e., public agency-affiliated, SOE-affiliated). Specifically, a VC is labelled as public agency- or SOE-affiliated when one of its 1^st^-level shareholders is classified as public agency or SOE, respectively. In the dataset, we also provided the information of equity ratio owned by each of the two sub-types separately, to facilitate studies on private-public interactions in the entrepreneurial financing context.

IVC: A VC is labelled as such when one of its 1^st^-level shareholders is classified as either professional or person.

CVC: A VC is labelled as such when one of its 1^st^-level shareholders is classified as corporate.

BVC: A VC is labelled as such when one of its 1^st^-level shareholders is classified as bank.

FVC: A VC is labelled as such when one of its 1^st^-level shareholders is classified as financial.

UVC: A VC is labelled as such when one of its 1^st^-level shareholders is classified as university.

PenVC: A VC is labelled as such when one of its 1^st^-level shareholders is classified as pension.

#### Mutually-exclusive VC type coding

For some specific research aims (such as computing VC syndication diversity)^1^, one may need to identify a VC with a unique affiliation. That is, a VC is classified to only one affiliation type and the typology of affiliation is mutually exclusive. To achieve this, we dedicatedly developed a mutually-exclusive coding scheme of VC type, following a sequential coding process as described below:

Considering the specific requirements for GVC and CVC in the literature, we firstly judged whether a VC is GVC; if not, then we decided whether it is CVC. Only if both answers were negative would we continue classifying it into other types. Specifically,

GVC: The criteria for GVC are distinct from other VC types because GVC is very complex in China and involves various forms of funding from public authorities who are in charge of government-guided funds, as well as SOEs including state-owned corporates, science or high-tech parks, and public accelerators. Following prior studies that classified GVC by dominant shareholders^7,13,14^, we classified a VC as GVC if it satisfies both of the following criteria: (1) at least one of its 1^st^-level shareholders is labelled as public agency or SOE, and (2) the total equity shares owned by public agency and SOE 1^st^-level shareholders are greater than that of any other type of 1^st^-level shareholders. In the dataset, we also provided the information of total equity ratio owned by state for those records. If only having the names of 1^st^-level shareholders but not their equity ratio data, we revised the criteria to be: (1) at least one of its 1^st^-level shareholders is labelled as public agency or SOE, and (2) the total number of public agency and SOE 1^st^-level shareholders is greater than that of any other type of 1^st^-level shareholders. If information of 1^st^-level shareholders is lacking, a VC is labelled as GVC if it is disclosed as a public agency or SOE from public sources.

CVC: Next, if a VC is not identified as GVC, we determined whether it is CVC using another standing-alone criterion, following prior CVC research^2,9^. As suggested by Chemmanur *et al*.^2^, CVCs are VCs with a unique corporate parent. Dushnitsky and Shaver^9^ further commented that CVC-backed ventures should not be spin-offs from the corporation. Consistent with their practices, we regarded a VC as CVC only if it has one unique 1^st^-level shareholder that is a corporate (which is denoted in the dataset as the CVC’s corporate parent), and made sure no ventures as spin-offs of the corporate in our sample. In some cases, a corporate owns 99% of equity of a VC and the remaining shareholders being subsidiaries of the corporate or person. For these cases we still classified the VC as CVC, and regarded the 99% corporate shareholder as the CVC’s corporate parent. If information of 1^st^-level shareholders is lacking, a VC is labelled as CVC if it is described by public sources as only affiliated with a corporate or if its main business is providing products and services other than VC investment (e.g., Google). As CVSource has recorded them as VC investors, there might be emerging CVC organizations within the corporations, and in these circumstances, we left their corporate parent as blank.

IVC, BVC, FVC, UVC, or PenVC: Lastly, if a VC does not belong to GVC or CVC, we determined its affiliation by the 1^st^-level shareholder type with the largest total equity, instead of the type of its absolute or relative controlling shareholder, because some shareholders may belong to the same type. Noteworthy, when a VC is owned by multiple corporates but not identified as CVC, we classified it as IVC, following the prior literature on CVC^2,9^. Likewise, if equity ratio data of 1^st^-level shareholders is lacking, we used the type with the largest number of shareholders. And if information of 1^st^-level shareholders is totally missing, a VC is accordingly labelled as IVC, BVC, FVC, UVC, or PenVC according to its main business revealed by public sources.

#### Step 3. Catch-all coding for remaining VCs

No matter whether we adopted non-exclusive or mutually-exclusive coding for VC type, when all the above procedures had been executed, we were still left with a small sample (66 VCs, 1% of the whole sample) that cannot be classified to any affiliation due to information unavailability. For these VCs, we used an additional catch-all rule and coded them as unknown VC.

#### Step 4. Coding for VC foreign background

On top of VC affiliation, we labelled a VC as foreign when all of its 1^st^-level shareholders are classified as foreign. If information of 1^st^-level shareholders is not available, we labelled a VC as foreign if it per se is registered in, or its brand originates from countries other than China. For each foreign VC, we supplied the information of its overseas location.

## Data Records

The VCAC dataset is available from figshare.com^15^. The VCAC dataset contains two plain text files with csv formatting, i.e., “VCAC.csv” and “Alias.csv”.

The “VCAC.csv” file encompasses the affiliation information of 6,379 VCs (in terms of VC_fullname) that have made investments in China between 2000 to 2016, and the total number of VC-year observations is 108,443. It should be noted that although the dataset seems to be a balanced panel data with every VC having 17 years of records, it is not the case in reality because VCs were established in different years. Nevertheless, a format of balanced panel data is to account for cases that in reality, some VCs may have invested (investment time based on CVSource) before they were formally founded (founding time based on Tianyancha.com). We further used a variable “Cross_sectional” to denote the inclusion of such cases, as explained below.

Variable definition of VCAC.csv is listed below:

1. VC_fullname: the full name of a VC, according to Tianyancha or Qichacha.
2. Year: a given year between 2000–2016.
3. GVCagency_ne: assigned the value of 1 if a VC is labelled as public agency-affiliated based on the non-exclusive VC type coding, and 0 otherwise.
4. GVCagency_equity: the total equity owned by public agency 1^st^-level shareholders in a VC. Noteworthy, missing value in this variable means that equity ratio data is not available.
5. GVCsoe_ne: assigned the value of 1 if a VC is labelled as SOE-affiliated based on the non-exclusive VC type coding, and 0 otherwise.
6. GVCsoe_equity: the total equity owned by SOE 1^st^-level shareholders in a VC. Noteworthy, missing value in this variable means that equity ratio data is not available.
7. IVC_ne: assigned the value of 1 if a VC is labelled as IVC based on the non-exclusive VC type coding, and 0 otherwise.
8. CVC_ne: assigned the value of 1 if a VC is labelled as CVC based on the non-exclusive VC type coding, and 0 otherwise.
9. BVC_ne: assigned the value of 1 if a VC is labelled as BVC based on the non-exclusive VC type coding, and 0 otherwise.
10. FVC_ne: assigned the value of 1 if a VC is labelled as FVC based on the non-exclusive VC type coding, and 0 otherwise.
11. UVC_ne: assigned the value of 1 if a VC is labelled as UVC based on the non-exclusive VC type coding, and 0 otherwise.
12. PenVC_ne: assigned the value of 1 if a VC is labelled as PenVC based on the non-exclusive VC type coding, and 0 otherwise.
13. GVC_excl: assigned the value of 1 if a VC is labelled as GVC based on the mutually-exclusive VC type coding, and 0 otherwise.
14. GVC_equity: the total equity owned by public agency-affiliated and SOE-affiliated 1^st^-level shareholders in a VC. Noteworthy, missing value in this variable means that equity ratio data is not available.
15. CVC_excl: assigned the value of 1 if a VC is labelled as CVC based on the mutually-exclusive VC type coding, and 0 otherwise.
16. Corporate_parent: the corporate parent for each CVC identified using the mutually exclusive coding method.
17. IVC_excl: assigned the value of 1 if a VC is labelled as IVC based on the mutually-exclusive VC type coding, and 0 otherwise.
18. BVC_excl: assigned the value of 1 if a VC is labelled as BVC based on the mutually-exclusive VC type coding, and 0 otherwise.
19. FVC_excl: assigned the value of 1 if a VC is labelled as FVC based on the mutually-exclusive VC type coding, and 0 otherwise.
20. UVC_excl: assigned the value of 1 if a VC is labelled as UVC based on the mutually-exclusive VC type coding, and 0 otherwise.
21. PenVC_excl: assigned the value of 1 if a VC is labelled as PenVC based on the mutually-exclusive VC type coding, and 0 otherwise.
22. Unknown: assigned the value of 1 if a VC is labelled as unknown VC, and 0 otherwise. For this variable, both non-exclusive and mutually-exclusive VC type coding generated the same.
23. Foreign: assigned the value of 1 if a VC is labelled as foreign VC, and 0 otherwise. For this variable, both non-exclusive and mutually-exclusive VC type coding generated the same.
24. Overseas_location: the overseas location of a foreign VC. If a foreign VC does not have a specific location (e.g., the International Finance Corporation), it is labelled as “International”. If no location information is available, it is labelled as “NA”. For this variable, both non-exclusive and mutually-exclusive VC type coding generated the same.
25. Cross_sectional: assigned the value of 1 if a particular VC-year observation satisfies either of the following conditions: (1) if we don’t have historical information for a VC (e.g., foreign VCs), we used the observation of 2016 to replace previous years, or (2) if a VC’s founding year is between 2000 and 2016 but it may have invested before its founding year according to CVSource, we used the observation of the founding year to replace previous years.

The “Alias.csv” file encompasses the VC names adopted by CVSource which are different from the full names appeared in public sources such as Tianyancha but the pair of two firms are essentially the same company. The total number of records is 1,116.

Variable definition of Alias.csv is listed below:

1. VC_fullname: the full name of a VC, according to Tianyancha or Qichacha.
2. Alias: the original name from CVSource.

## Technical Validation

We took the following measures to ensure the quality of the dataset.

### Upon dataset composition: Matching multiple datasets

To start, we relied on CVSource database to generate a list of 6,553 VCs who have invested in China between 2000 and 2016. We then used their names to search in public sources including Tianyancha and Qichacha to collect the information of their 1^st^-level shareholders. This step was initially done by machine and algorism, deriving 28,968 unique 1^st^-level shareholders; however, VC names from CVSource could be abbreviations or former names that cannot be perfectly matched with those full names in public sources, making it problematic to solely reply on algorism. To address this issue, we manually double-checked and corrected any inconsistency to ensure that each VC name from CVSource is aligned with the name we collected from public sources. We also checked any duplications in VC names because the CVSource database may record a former and a current name of a VC but treat them as two different VCs. Finally, we derived 22,757 unique 1^st^-level shareholders for 6,379 unique VCs.

### Upon coding of shareholder type: Triangulating across sources and coders

The coding of shareholder type was divided into three parts and conducted completely by human coders, following the three-layered coding scheme. The first part dealt with the Layer-I coding on shareholder business scope, i.e., applying the typology developed and distinguishing between the nine types of shareholders. It involved substantial manual check against information about business scope recorded in public sources. However, information about business scope from different sources may be inconsistent or contradictory. For example, the information about business scope recorded by Tianyancha could be different from the information of “about us” or “history” listed on firms’ official websites. In addition, the registered business scopes on Tianyancha may be too extensive to code. To ensure the quality of this part, we developed a procedure of cross-checking and recruited 17 senior students and master students from two famous universities in China as part-time research assistants. The first two authors trained the research assistants, and gave them training tasks to finish until they passed our checks. The research assistants were then assigned to classify the type of 1^st^-level shareholders, and the second and third authors double-checked all the results manually by repeating the whole procedure. If there was any disagreement between a research assistant and the second and third authors, the first author was involved to give independent opinion and then invited the team to discuss, in order to achieve consent.

The second part addressed the Layer-II coding on SOE nature. As aforementioned, this classification involves multiple levels of equity penetration to trace the ultimate nature of state-ownership, which task is not able to be done by machine. SOEs include central and local ones. For central SOEs, the Stated-owned Assets Supervision and Administration Commission of the State Council (SASAC) provides a directory of 97 central SOEs in China. However, we did not find a similar list for the local SOEs. To solve this issue, we consulted experts from VC industry and obtained four SOE classification criteria from different sources (e.g., NPC, SASAC, Ministry of Finance, China Security Regulatory Commission, National Bureau of Statistics). We chose to follow the majority opinion and used the SOE definition stated in the “Measures for the Supervision and Administration of the Transactions of State-owned Assets of Enterprises (Order No. 32)” and “Measures for the Supervision and Administration of State-owned Equity of Listed Companies (Order No. 36)”. Therefore, we collected related information from multiple sources including the directory of central SOEs, subsidiaries of the central SOEs, Baidu, Google, Tianyancha, firm websites, etc. Since the Layer-II coding on SOE nature is not mutually exclusive with Layer-I coding on shareholder business scope, to further ensure the quality, the third author not only coded on Layer-II SOE nature but also further checked the Layer-I coded results for shareholders that were classified as SOE type. The second author was then invited to go through the work done by the third author again. If there was any disagreement between the third author and second author, the whole team were involved in discussion to achieve consent.

The third part was to conduct the Layer-III coding on foreign background. This part is difficult because some foreign VCs do not have their official websites, and the information about their locations could be inconsistent across public sources such as Bloomberg and Crunchbase. In some cases, two distinct VCs from different countries may share the same name, which created further confusion. To systematically enhance the quality of this part, the second author used Google to compile information from various sources (e.g., media reports, IPO prospectus) and compared with the investment records in CVSource until information was saturated. If the judgement was not obvious, the first and third authors were invited to give independent opinion and make a final decision.

### Upon coding of VC type: Comparing with prior studies

Next, we combined our VCAC dataset (using VC affiliation information) with the CVSource dataset (using investment deal information) to further validate the VCAC dataset by comparing with prior studies that reported similar attributes of VCs in China. The following validations merged VC affiliation data from VCAC.csv and investment deal information from CVSource, based on VC-year level.

**Fig. 1:**
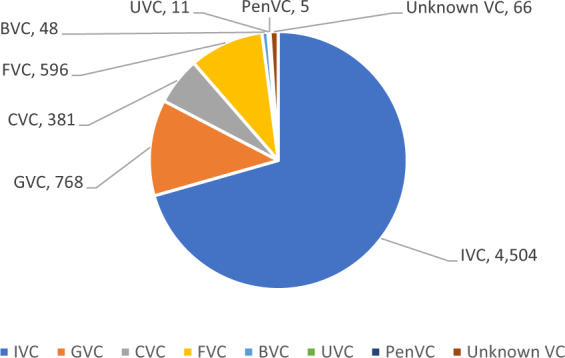
Number of VCs by affiliation. This figure reports the distribution of 2016’s affiliation for all VCs that had investments in China between 2000 and 2016.

Figure 1 shows the distribution of VCs by affiliation in China during 2000–2016, if adopting the mutually-exclusive coding of VC type. Since a VC’s affiliation type may vary across years (213 out of 6,379 VCs having changes in affiliation types across years), the data of 2016 was adopted to generate this figure. In terms of the number of VCs, the largest group is IVCs (70.61%), followed by GVCs (12.04%), FVCs (9.34%), and CVCs (5.97%). The number of BVCs, UVCs, and PenVCs are almost neglectable in China.

**Fig. 2:**
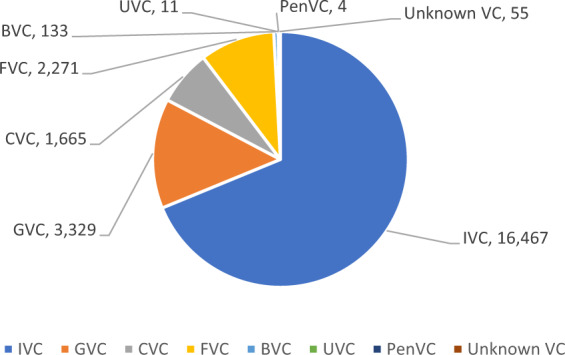
Number of VC investments by affiliation. This figure reports the distribution of VC affiliation for all investment deals in China between 2000 and 2016.

Figure 2 shows the distribution of VC investments by affiliation in China during 2000–2016, if adopting the mutually-exclusive coding of VC type. As shown in Fig. 2, during the period of 2000–2016, IVCs invested the most (68.8%) in China’s market, greater than GVCs (13.91%), CVCs (6.96%), and FVCs (9.49%), while BVCs, UVCs, and PenVCs contributed less than 1% due to the small numbers of these VCs.

**Fig. 3:**
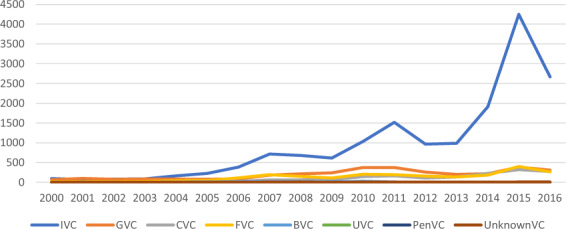
Number of VC deals by affiliation and year. This table reports the number of investment deals made by VCs that have invested in China between 2000 and 2016, by year and type of VC affiliation.

Figure 3 shows the annual trend of investment deals made by each type of VC, if adopting the mutually-exclusive coding of VC type. As shown in Fig. 3, the number of investment deals kept climbing for each type of VCs and has reached a peak in 2015 due to China’s policy advocating mass innovation and entrepreneurship, i.e., “*Shuangchuang*” in Chinese.

Next, we compared our dataset with prior studies that collected secondary data and conducted careful manual check on VC affiliation because, as explained, the existing datasets about China’s VC affiliation are either lacking or with low quality. We were aware of two streams of such studies.

The first stream of prior studies focused on GVC in the context of China. For example, Brander *et al*.^14^ collected a list of VCs from VentureXpert database during the 2000–2008 period and manually coded whether a VC is GVC by referring to information from Capital IQ and websites of VCs. Among their data of VC activities in 25 countries, the sample about China showed that 23% of investment deals were invested by GVCs. We restricted our sample to the same time period, and our data showed that during 2000–2008, the percentage of GVC investment deals is 22.01%. The difference between Brander *et al*.^14^ and ours could be attributed to the difference in GVC classification criteria. Specifically, Brander *et al*.^14^ excluded international government institutions, nor did they count for GVCs that are partially state-owned. In addition, our criteria are more contextualized to China. For example, we followed No. 32 and No. 36 in China to classify Chinese SOE shareholders for a VC, while no information is available on how Brander *et al*.^14^ counted such shareholders. Therefore, it is acceptable that our percentage of GVC investment deals is slightly different from theirs.

We also compared with studies that used China’s VC datasets. For example, Zhang^7^ sampled VC investments in China in the 1995–2011 period, using data from PEdata but with manual correction on GVC classification. Columns 2–3 of Table 1 cited Zhang’s distribution of investment deals by different types of VC investment, and column 4–5 of Table 1 reported corresponding distributions based on our dataset. Both Zhang’s sample and ours covered 16 years, with ours being delayed by five years. The distributions between the two samples are quite consistent.

**Table 1:** Comparison with prior literature on GVC investments.

Type of investments	# in Zhang’s sample 1995–2011^7^	% in Zhang’s sample 1995–2011^7^	# in our sample 2000–2016	% in our sample 2000–2016
GVC-alone	611	11.70%	2,548	10.65%
GVC-only syndication	47	0.90%	217	0.91%
Private VC-alone	3,265	62.52%	14,866	62.11%
Private VC-only syndication	1,005	19.25%	5,031	21.02%
GVC-private VC mixed syndication	294	5.63%	1,274	5.32%
Total	5,222	100%	23,936	100.00%

The second to third columns cite the distributions based on Zhang^7^. The last two columns report corresponding distributions based on our dataset.

Another stream of prior studies focused on CVC. However, we were not able to spot any notable CVC research in the context of China. Therefore, we compared our results with CVC studies conducted in the context of U.S., with an assumption that the trends of CVC development in these countries could be parallel. For example, Wang and Wan^16^ connected Thomson Financial’s Securities Data Corporation (SDC) New Issue database with VentureXpert database, and sampled 200 VC-backed IPOs from 2000 to 2007. They generated two measures relating to VC type. They calculated IVC ownership as the equity ratio held by IVCs divided by the total equity share of VCs in a firm, and computed CVC ownership as the same but held by CVCs. According to their descripitives, the average of IVC equity ratio is 72.3%, and that of CVC is 9%. Although their measures are based on equity share and are different from ours that counted the number of deals, the relative size is comparable with our results (IVC deal percentage 68.8%, and CVC deal percentage 6.96%).

Besides VC affiliation, we also compared with prior studies to cross-check the quality of our labelling on foreign VC. Prior studies on foreign VCs mainly relied on existing databases such as PEdata or CVSource (and the criteria of those datasets regarding foreign VC were not disclosed). For example, based on PEdata, Wang and Wang^17^ collected a sample consisting of 495 identifiable VC investments made by 84 foreign VCs from 1999 to 2006, and the annual number of VC investments by foreign VCs is reported in the second column of Table 2. Likewise, we collected such data from CVSource, used CVSource’s own label on foreign VC, and summed up the annual number of VC investments by foreign VCs, as shown in the third column of Table 2. Lastly, we used our label of foreign VC, combined with CVSource data of deal records but selected the overlapped years with Wang and Wang^17^, and recalculated the annual number of VC investments by foreign VCs, as shown in the last column of Table 2. The results indicate that while our label of foreign VC is independent from CVSource’s, our results are quite close to theirs. However, we did find significant difference between Wang and Wang^17^ and CVSource’s results. This could be attributed to the difference in deal record data between PEdata and CVSource. Noteworthy, our dataset of VC affiliation and other attributes is independent from any VC investment databases, and can be linked with any of them for research needs.

**Table 2:** Comparison with prior literature on the number of foreign VC investments.

Funding year	# in Wang and Wang’s sample using PEdata^17^	# in CVSource	# in our sample
2000	31	37	46
2001	44	40	35
2002	18	35	36
2003	21	52	45
2004	40	121	108
2005	79	162	162
2006	113	290	286
Total	346	737	718

The second column reports the summary from Wang and Wang^17^ using PEdata. The third column reports the summary based on records made by CVSource. The fourth column reports the summary based on our coding results.

## Usage Notes

The csv files are in UTF-8 and are comma separated. VC name is in Chinese.

In future, we will keep maintaining the dataset. For example, we may add new features/variables to this dataset when theoretically meaningful (either emerging from our future research or suggested by dataset users from external). If so, a log file will be available for users to understand any new features.

Box 1 Stata pseudocode to determine non-exclusive VC type. gen Dum_GVCagency = 0

replace Dum_GVCagency = 1 if strmatch(sh_type_new, “*Agency*”)

gen Dum_GVCsoe = 0

replace Dum_GVCsoe = 1 if strmatch(sh_type_new, “*SOE*”)

gen Dum_CVC = 0

replace Dum_CVC = 1 if strmatch(sh_type_new, “*Corporate*”)

gen Dum_IVC = 0

replace Dum_IVC = 1 if strmatch(sh_type_new, “*Professional*”)

replace Dum_IVC = 1 if strmatch(sh_type_new, “*Person*”)

gen Dum_BVC = 0

replace Dum_BVC = 1 if strmatch(sh_type_new, “*Bank*”)

gen Dum_FVC = 0

replace Dum_FVC = 1 if strmatch(sh_type_new, “*Financial*”)

gen Dum_UVC = 0

replace Dum_UVC = 1 if strmatch(sh_type_new, “*University*”)

gen Dum_PenVC = 0

replace Dum_PenVC = 1 if strmatch(sh_type_new, “*Pension*”)

gen Dum_Unknown = 0

replace Dum_Unknown = 1 if strmatch(sh_type_new, “No”)

gen Dum_Foreign_shareholder = 0

replace Dum_Foreign_shareholder = 1 if strmatch(sh_type_new, “*Foreign*”) & sh_name!=””

gen Dum_Foreign = 0

replace Dum_Foreign = 1 if countSh==0 & strmatch(sh_type_new, “*Foreign*”)

replace Dum_Foreign = 1 if countSh==1 & Dum_Foreign_shareholder == 1

bys SVC_name year: egen min_Foreign_shareholder=min(Dum_Foreign_shareholder)

replace Dum_Foreign = 1 if countSh>1 & min_Foreign_shareholder == 1

foreach var in Dum_GVCagency Dum_GVCsoe Dum_CVC Dum_IVC Dum_BVC Dum_FVC Dum_UVC

Dum_PenVC Dum_Unknown {

                    bysort VC_name: egen ‘var’_ne = max(‘var’) }

renvars Dum_GVCagency_ne-Dum_Unknown_ne, predrop(4)

duplicates drop VC_fullname, force

## Data Availability

The program to calculate and determine VC affiliation is Stata 15.0. Box 1 demonstrates a pseudocode of Stata when coding the VC type in a non-exclusive way.
